# Effect of Sonic Agitation of a Binary Mixture of Solvents on Filling Remnants Removal as an Alternative to Apical Enlargement—A Micro-CT Study

**DOI:** 10.3390/jcm9082465

**Published:** 2020-08-01

**Authors:** Inês Ferreira, Pedro S. Babo, Ana Cristina Braga, Manuela E. Gomes, Irene Pina-Vaz

**Affiliations:** 1 Faculty of Medicine, University of Porto, 4200-319 Porto, Portugal; 2 CINTESIS, Faculty of Dental Medicine, University of Porto, 4200-393 Porto, Portugal; 3 3B’s Research Group, I3Bs-Research Institute on Biomaterials, Biodegradables and Biomimetics, University of Minho, Headquarters of the European Institute of Excellence on Tissue Engineering and Regenerative Medicine, AvePark, Parque de Ciência e Tecnologia, Zona Industrial da Gandra, Barco, 4805-017 Guimarães, Portugal; pedrombabo@gmail.com (P.S.B.); megomes@dep.uminho.pt (M.E.G.); 4 ICVS/3B’s – PT Government Associate Laboratory, 4805-017 Braga/Guimarães, Portugal; 5 Department of Production and Systems, ALGORITMI Center, University of Minho, Campus de Gualtar 4710-057 Braga, Portugal; acb@dps.uminho.pt

**Keywords:** EndoActivator, gutta-percha, micro-CT, retreatment, sealer, solvents

## Abstract

Background: This work aimed to evaluate the efficacy of sonic agitation of a binary mixture of solvents (methyl ethyl ketone/tetrachloroethylene) on filling remnants removal and compare the effects of solvent agitation with the enlargement to the next instrument size. Methods: Twenty-four mandibular incisors were prepared with ProTaper Next (X1, X2) and obturated with the single-cone technique and AH Plus sealer. The teeth were retreated with ProTaper Universal Retreatment and ProTaper Next and divided into two groups (*n* = 12) according to the final instrument (X3 or X4). All canals were submitted to a supplementary procedure consisting of a mixture of solvents―methyl ethyl ketone/tetrachloroethylene, agitated with EndoActivator. The volume of filling remnants was assessed through micro-computed tomography in the apical 5 mm. Statistical analysis was performed with a significance level of 5%. Results: The supplementary procedure of agitation of the solvent mixture was beneficial in both groups (*p* < 0.05). There were no statistically significant differences between canals re-prepared until X4 and canals re-prepared until X3 plus solvent (*p* > 0.05). Conclusions: An additional step with a two-solvent solution potentiated by EndoActivator showed to be very effective for the removal of gutta-percha and resinous sealer remnants from apical root canals of mandibular incisors, avoiding further enlargement.

## 1. Introduction

Non-surgical endodontic retreatment is challenging and, although there have been advancements in instrumentation technology, the predictability of filling material removal is still low. Strategies for cleaning and disinfection will essentially depend on either supplementary approaches or enlargement to even larger sizes [1,2,3,4]. Adjunctive procedures show controversial outcomes and still do not enable the thorough cleaning of root canals [5,6,7,8,9,10]. Using a larger preparation size would be expected to result in fewer unprepared areas and, consequently, a higher volume of old filling material removal, optimizing disinfection [7,10,11,12,13]. Nevertheless, a correlation between disinfection and optimal canal size was failed to be proved [14,15].

Concerns about the cytotoxic potential of using solvents and the associated risk of hindering the cleaning process of root canal walls and isthmuses made them fall into disuse [8,16,17,18]. The efficacy of solvents for retreatment purposes has been assessed in micro-computed tomographic studies (micro-CT), but the results are inconclusive [8,19,20,21].

Previous research suggested a non-traditional solvent-methyl ethyl ketone (MEK)―for dissolving the AH Plus sealer [22] and tetrachloroethylene for gutta-percha [23], both potentiated by agitation, as an additional and final step in the removal of obturation material remnants. We hypothesize that the binary mixture of solvents might have a complementary dissolution advantage, aside from the easiness of being a single-step procedure, thus overcoming the limitations of re-preparation in the removal of the materials left behind and preventing weakening of the teeth.

No study has evaluated if any adjunctive procedure would result in a filling removal similar to that of larger instrumentation. Therefore, this work aimed to evaluate through micro-CT analysis the efficacy of sonic agitation of a mixture of solvents (MEK/tetrachloroethylene (1:1)) on filling material remnant removal and compare the effects of the additional solvent agitation with the enlargement to the next instrument size.

## 2. Experimental Section

### 2.1. Specimen Selection

After approval by the Ethics Committee of the Faculty of Dental Medicine of the University of Porto (nº3/2018), the teeth were selected from a group of 100 mandibular central single-rooted incisors extracted for reasons unrelated to this study. Mesiodistal and buccolingual radiographs were taken, and teeth with a single canal (Vertucci type 1) were selected. Each tooth was then inspected under an operating microscope, and all teeth with root fractures, internal or external root resorptions, and immature apex were excluded. The teeth were gauged using a stainless-steel K-file (Dentsply Sirona, Ballaigues, Switzerland), and only those where the first instrument to bind at working length was a size 15 were selected. Twenty-four teeth were selected according to these criteria.

### 2.2. Root Canal Preparation and Filling

Teeth were stabilized during the preparation procedures. A size 10 K-file was used to determine the working length (WL), which was about 1 mm shorter than the full root-canal length. The canals were prepared with ProTaper Next (X1, X2), and repeatedly irrigated using 3% NaOCl (30G needle-Max-I-Probe, Dentsply International, Inc., York, PA, USA) after each file (a total of 5 mL of NaOCl was used). The apical patency was checked with a size 10 K-type file. The final rinse was performed with 2 mL of 3% NaOCl followed by 2 mL of 17% EDTA for 1 min, both sonically agitated for 1 min (three periods of 20 s) with EndoActivator (Dentsply Sirona, Ballaigues, Switzerland, (size 25.04 taper and 10,000 cycles/min; 2 mm short of the WL)) and, finally, 1 mL of distilled water. After drying with paper points, the root canals were filled with ProTaper Next X2 gutta-percha cone (Dentsply Sirona, Ballaigues, Switzerland) and AH Plus Jet (Dentsply DeTrey, Konstanz, Germany), using the single-cone technique. The sealer was placed using a lentulo spiral filler. The roots were radiographed in buccolingual and mesiodistal directions to ensure consistency of the root filling procedure. Teeth in which the root canal filling was judged unsatisfactory were replaced by new samples. The access cavities were sealed with a temporary restorative material (IRM, Dentsply Sirona, Ballaigues, Switzerland), and the samples were stored in 100% humidity at 37 °C for 2 weeks.

### 2.3. Retreatment and Re-Preparation Procedures

Retreatment was performed with ProTaper Universal Retreatment (D1, D2, and D3) and Hedström files (Dentsply Sirona, Ballaigues, Switzerland). ProTaper files D1, D2, and D3 were used for the removal of the bulk of filling material at the coronal, middle and apical thirds of the root canals, respectively. The files were removed from the canal, cleaned with a sponge, and placed again until the required length was achieved. For the refinement of the residual filling material removal, or whenever necessary to reach the WL, Hedström files of size 30 and 25 were used with brushing or circumferential quarter-turn push-pull filling motions, allowing better control of the cleanliness of root walls. Patency was checked with a size 15 K-type hand file. A total of 15 mL of 3% NaOCl was used.

The filling materials were considered to be completely removed when no remaining filling materials were observed inside the root canal, on the ProTaper Next or Hedström instrument, or floating in the irrigation solution, with the help of a microscope (OPMI Pico, Carl Zeiss, Jena, Germany).

Teeth were randomly allocated into two groups of 12 specimens:

- Group 1: Re-preparation with ProTaper Next X3 file
- Group 2: Re-preparation with ProTaper Next X3 and X4 files

Teeth were prepared following the same protocol used in the instrumentation of the canals, using a total of 5 mL of 3% NaOCl, and a final irrigation was performed with 2 mL of 3% NaOCl, followed by 2 mL of 17% EDTA, both activated with EndoActivator for 1 min.

### 2.4. Supplementary Procedure

All specimens were submitted to a supplementary procedure consisting of using a binary mixture of solvents―MEK/tetrachloroethylene (1:1) (VWR International SAS, Fontenay-sous-Bois, France), sonically agitated. Each canal was rinsed with a total of 1 mL of the solvent mixture agitated sonically for 5 min using the EndoActivator (red tip, 25.04 at a speed of 10,000 cycles/min with “in-and-out” movements; 2 mm short of the WL). The mixture was refreshed after each minute. Lastly, all the canals were irrigated with 5 mL of distilled water to neutralize the action of the mixture of solvents.

### 2.5. Micro-CT Imaging Analysis

The samples were scanned at three moments: After root canal filling, after conventional retreatment/re-preparation, and after the supplementary procedure (solvent/EndoActivator), using a high-resolution micro-CT (Skyscan 1272, Bruker, Kontich, Belgium). A series of two-dimensional projections of the specimens with a resolution of 10 μm were acquired by irradiation with penetrative X-rays and using a 0.5-mm-thick aluminum filter. This acquisition was made over a rotation range of 360° with a rotation step of 0.45°. Data were reconstructed using the software NRecon (version 1.7.1.0, Bruker, Kontich, Belgium) and analyzed on a CT analyzer (version 1.17.0.0, Bruker, Kontich, Belgium). The region of interest (ROI) was defined as a circle centered in the root canal. For standardization, the apical region was analyzed between 1 and 5 mm above the apex opening. An auto-interpolation of the manually defined ROI was conducted to obtained volumes of interest (VOI) corresponding to the apex regions of 1–3 mm and 3–5 mm, which were the essential basis for the quantitative analyses. The three-dimensional models were generated using the software CT Vox (version 3.3.0 r1412, Bruker, Kontich, Belgium).

**Figure 1:**
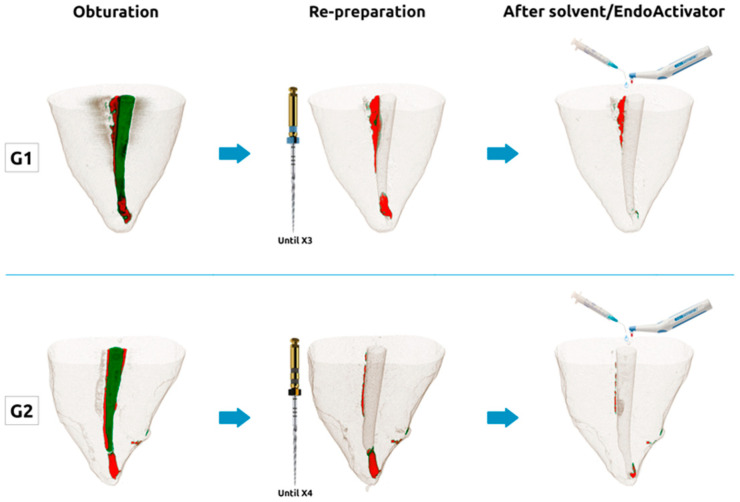
Micro-computed tomographic (Micro-CT) images of representative specimens subjected to the different procedures (obturation with X2 gutta-percha cone and AH Plus sealer (G1 and G2); re-preparation until X3 (G1) and X4 (G2); after solvent/EndoActivator (G1 and G2). Green: Gutta-percha; red: Sealer.

All specimens were prepared by a single operator, following standard clinical endodontic practices, and the procedures are schematically illustrated in Figure 1. Each instrument was used in three teeth and then discarded.

### 2.6. Statistical Analysis

The sample calculation was performed using the G*Power software, version 3.1.9.6 (Franz Faul, Universität Düsseldorf, Düsseldorf, Germany) [24]. The data from a previous study [25] on retreatment using single-rooted teeth were used to determine the effect size for the present study (i.e., 1.80). For unilateral testing, an alpha-type error of 0.05, a beta power of 0.95, and a ratio of N2/N1 = 1 were also stipulated. A family of tests used for the differences in means between two independent groups indicated eight specimens per group as the ideal size. Considering 12 specimens per group, the power increases to 0.996.

The Kolmogorov–Smirnov (KS) test was applied to test for data normality. Based on the KS results, statistical analysis to assess differences between groups was performed using the Student’s *t*-test or the Mann–Whitney test, depending on whether normality was verified or not, respectively. A paired *t*-test was used to compare the volume of filling material remnants in the samples prepared by each technique. For all statistical tests, the level of significance was established at 5% (*p* < 0.05).

## 3. Results

Table 1 shows the mean and standard deviation values of the filling materials at the different root sections. The t-test confirmed a similar volume of filling materials before retreatment between groups and sections (*p* = 0.385 for 1–3 mm; *p* = 0.496 for 3–5 mm; *p* = 0.417 for 1–5 mm), which allowed the comparison between groups.

A significant reduction in the filling materials after re-preparation was detected in the two groups for both the assessed regions. Group 1 (re-preparation until X3) presented more remnants than Group 2 (re-preparation until X4), both for the sections and the full apical canal, without statistically significant differences (*p* = 0.200 for 1–3 mm; *p* = 0.136 for 3–5 mm; *p* = 0.087 for 1–5 mm).

There were also no significant differences between Group 1 and 2 after using the solvent (*p* = 0.974 for 1–3 mm; *p* = 0.622 for 3–5 mm; *p* = 0.412 for 1–5 mm). However, both groups showed significant differences (*p* < 0.05) in the amount of remnants from re-preparation (until X3 or X4) to after solvent exposure. Thus, the supplementary procedure with the application of the mixture of solvents was beneficial in both groups. Additionally, no statistically significant differences were found between re-preparation until X4 before solvent (Group 2) and re-preparation until X3 after solvent (Group 1) (*p* = 0.073 for 1–3 mm; *p* = 0.959 for 3–5 mm; *p* = 0.358 for 1–5 mm).

## 4. Discussion

The present investigation aimed to study the effectiveness of the sonic agitation of a mixture of solvents on the volume of filling material remnants in mandibular incisors, and to assess if it would compare to progressive apical enlargement. Similar to other investigations, the sample was standardized, based on an initial radiographic evaluation and apical gauging, to avoid relevant bias regarding canal anatomy and filling material volume [11,12]. Statistical analysis revealed a similar volume of filling materials before retreatment and between groups and sections (Table 1).

The apical third of the root canal is considered the most critical zone for thorough disinfection due to its anatomical complexities and persistence of accumulated debris and bacterial biofilms [26,27]. Thus, filling materials were mapped in the apical 5 mm using micro-CT after root canal filling, after mechanical re-preparation, and after the adjunctive final solvent protocol [7]. The volume of the residual filling material was calculated for the full apical canal (5 mm from the apex) and the different Sections 1–3 and 3–5 mm from the apex [8].

Patency was re-established in all root canals, but in around 60% of the samples, this was only after solvent exposure. The enlargement from an X3 to an X4 ProTaper Next file did not statistically significantly decrease the volume of filling remnants, both in the full apical canal and in the 1–3 mm and 3–5 mm sections. However, the solvents’ mixture applied (MEK/tetrachloroethylene), agitated by a sonic device (EndoActivator), significantly decreased filling remnants. Root canals were cleaner after solvent exposure than without solvent, both with the X3 and the X4 re-preparation (Figure 1 and Table 1). Additionally, root canals prepared to a larger size (size 40) were as clean as smaller preparations (size 30) after the sonically agitated solvent mixture. Accordingly, it was recently reported that when sodium hypochlorite was ultrasonically activated, smaller preparations resulted as clean as larger preparations [28]. Even though the referenced study evaluated the remaining pulp tissue and debris instead of residual filling materials, the results demonstrate the influence of the irrigation strategy overcoming the insufficiencies of the instrumentation approach, irrespective of the canal configuration (round vs. oval). Further research should focus particularly on the effect of different apical preparation sizes in conjunction with activated irrigation and whether it would result in similar filling removal [12,28].

An increased enlargement of apical re-preparation has been associated with a lower volume of residual filling materials [7,10,12]. This difference has been reported between additional instrumentation with larger tip sizes (size 35–40) compared to size 25. In the present study, the results showed that an increase to the next instrument size (tip size 30 to 40) might not directly correspond to a decrease in residual obturation material volume. Different methodologies, tooth types, instrument sizes, and motions can influence the shaping ability of the instruments [1,4,6,29,30,31,32]. Even though the number of unprepared areas was shown to be inversely proportional to the increase of each preparation size, a consequent thinner remaining dentin can compromise the tooth’s survival [11,28,33]. Additionally, a larger apical size did not result in a complete apical preparation, but it could lead to unnecessary removal of dentin, with the risk of iatrogenic procedures [34,35]. Filling remnants remaining in untouched recesses and isthmus might prevent irrigants and dressings from reaching biofilms, not allowing for complete root canal wall exposure. An additional step with an effective solvent for both filling materials should improve disinfection, by further removing potentially infected filling remnants and avoiding unnecessary enlargement or risk of procedural errors. Besides the improved dissolution efficacy reported, the agitation should promote better solvents’ dispersion, reaching areas usually inaccessible to endodontic instruments, and thus improving root canal cleanliness [6,36,37].

Recently developed finishing files, such as XP-endo Finisher R (FKG Dentaire, La Chaud-de-Fonds, Switzerland), have been highlighted as an effective supplementary approach. However, they are advised to be used following preparations with apical diameters of 30 or more [7,10,38]. New design ultrasonic tips have also shown good performance, but, again, the additional removal of dentine may be a disadvantage [39].

Previous studies found no differences in the volume of obturation materials removed with the use of traditional endodontic solvents, independently of ultrasonic agitation (PUI) [8,20,21]. AH Plus is an epoxy resin-based sealer commonly used due to its adequate properties as an endodontic sealer [40]. Targeting the sealer chemistry proved to be crucial in the achievement of solvent efficacy for retreatment purposes [22,41]. These results may be explained, firstly, by the specificity of the solvents in the binary mixture―MEK and tetrachloroethylene—which were described to be effective for epoxy-resin sealer and gutta-percha, respectively. Their efficacy, enhanced by ultrasonic agitation, was reported to be similar to the traditional chloroform, without its potential cytotoxicity [22,23]. The 1:1 mixture would enable a clinical single-step procedure since MEK proved to act mainly on resinous sealer dissolution, while tetrachloroethylene could have an additional effect on the sealer besides gutta-percha dissolution [23]. We opted to use the EndoActivator for the additional cleaning with agitation due to its clinical safety and easiness of use [42]. Sonic and ultrasonic devices have been reported to be equivalent in the removal of filling materials when activating sodium hypochlorite [6]. To our knowledge, there are no reports about the efficacy of sonic agitation of solvents on filling materials removal.

Even though not all the samples were completely free from remnants, due to the time-dependent dissolution effect [22,23], it can be expected that the longer the period of agitation, the better the cleanliness achieved in complex root canal anatomies. Due to the low cytotoxicity profile of the suggested solvents in the mixture, as compared to the traditional chloroform, damage to the dentine or other relevant cytotoxic hazards are not expected, even with longer periods [43,44,45]. Nevertheless, all procedures must follow the gold standard quality guidelines for endodontic treatment [46]. Even though there seems to be a consensus on the use of solvents in the early stages of instrumentation, due to reaching the working length in a shorter time, studies confirming their advantages are still scarce [2].

One of the drawbacks of the present ex-vivo investigation was the lack of matched-pair design by micro-CT [47]. However, recent investigations using pair-matched root canals seem to corroborate the present results, highlighting the role of irrigant agitation in overcoming the insufficiencies of mechanical instrumentation [28]. Further studies that control these potentially confounding effects and assess other tooth anatomies should confirm the present findings.

Overall, our findings suggest that a non-traditional binary mixture of solvents―MEK/tetrachloroethylene (1:1)—potentiated by sonic agitation, as herein proposed, holds the potential to become an additional and final step in retreatment procedures, complementing mechanical cleaning in the removal of the obturation material. It could be used in a single solution with a dual objective of dissolving gutta-percha and a resinous sealer and is presented as an alternative to chloroform without its potential hazards. The strategy proposed might enable the optimization of retreatment procedures to obtain cleaner canals without further enlargement.

## Figures and Tables

**Table 1:** Volume (mm^3^) of filling material in different sections (1–3 mm and 3–5 mm) of the apical portion (1–5 mm). (G1: Canals re-prepared until X3 file; G2: Canals re-prepared until X4 file).

	Obturation			Re-Preparation			After Solvent/EndoActivator		
	G1	G2	Total	G1	G2	Total	G1	G2	Total
**1–3 mm**	0.260 (± 0.041)	0.246 (± 0.035)	0.252 (± 0.038)	0.051 (± 0.035)	0.036 (± 0.022)	0.043 (± 0.029) ^a^	0.018 (± 0.024)	0.013 (± 0.014)	0.015 (± 0.019) ^a^
**3–5 mm**	0.483 (± 0.077)	0.453 (± 0.124)	0.468 (± 0.103)	0.057 (± 0.050)	0.030 (± 0.031)	0.043 (± 0.043) ^b^	0.030 (± 0.033)	0.019 (± 0.022)	0.024 (± 0.028) ^b^
**1–5 mm**	0.743 (± 0.104)	0.699 (± 0.146)	0.720 (± 0.127)	0.108 (± 0.065)	0.066 (± 0.048)	0.086 (± 0.059) ^c^	0.047 (± 0.047)	0.031 (± 0.029)	0.039 (± 0.039) ^c^

^a,b,c^―*t*-test revealed significant differences *p* < 0.05.
